# Compact on-chip fluorescence microscope for dynamic imaging of cellular processes and biomimetic systems

**DOI:** 10.1088/2515-7647/ae6ffe

**Published:** 2026-06-25

**Authors:** Somaiyeh Khoubafarin, Aakash Subramanian, William Daniel Gorgas, Cyrus Koogan, Indira Sigdel, MD Alamgir Kabir, Yuan Tang, Aniruddha Ray

**Affiliations:** 1 Department of Physics and Astronomy, University of Toledo, Toledo, OH 43606, United States of America; 2 Department of Chemistry and Biochemistry, University of Toledo, Toledo, OH 43606, United States of America; 3 Department of Electrical Engineering and Computer Science, University of Toledo, Toledo, OH 43606, United States of America; 4 Department of Bioengineering, College of Engineering, University of Toledo, Toledo, OH 43606, United States of America

**Keywords:** on-chip fluorescence microscope, biomimetic systems, CMOS sensor

## Abstract

Real-time and High-throughput fluorescence imaging is essential for probing dynamic cellular behavior in biomimetic and tissue-on-chip systems. While fluorescence microscopy provides high sensitivity and subcellular resolution, the intrinsic heterogeneity of these engineered tissues requires imaging multiple regions to obtain representative biological information. Meeting this need typically demands mechanical scanning systems, which add both hardware and software complexity and substantially increase cost. Here, we developed a compact and low-cost on-chip fluorescence microscopy platform that integrates a two-dimensional microlens substrate directly onto a complementary metal-oxide-semiconductor sensor to achieve high resolution and signal-to-noise ratio (SNR). Excitation light is delivered laterally through a prism to induce total internal reflection, effectively rejecting background illumination and allowing only fluorescence emission to reach the detector. The system’s optical geometry was optimized using analytical modeling and numerical simulations to maximize photon collection and SNR. Using this optimized system, we first demonstrated its capability at the cellular level by capturing drug-induced oxidative stress and rapid intracellular signaling dynamics in BT-20 breast cancer cells. Once we were successfully able to monitor these dynamic processes, we next applied the system to biomimetic models. In a microfluidic tumor-endothelial co-culture, we visualized the passage of small molecules, nanoparticles, and ions across the endothelial barrier under controlled flow conditions. Integration with microfluidic co-culture systems further demonstrated its ability to study complex interactions within tumor microenvironments. By combining simplicity, sensitivity, and compatibility with biomimetic platforms, this on-chip fluorescence microscope enables wide-field monitoring of cellular dynamics across large populations, allowing observation of cell-to-cell variability and supporting applications in drug screening, cellular signaling studies, and tissue-on-chip research.

## 1 Introduction

Real-time monitoring of cellular processes and interactions in biomimetic systems, such as tissue-on-chip models, is crucial for getting insights into physiological responses and disease mechanisms [1, 2]. Biomimetic systems replicate the architecture and functionality of human tissues, thereby reducing the reliance on animal testing, which can be costly, time intensive and ethically challenging [3, 4]. For instance, in cancer research, tissue-on-chip models can simulate tumor microenvironments (TME), enabling the study of cancer cell behavior, drug delivery and its effects [5]. This approach not only enhances the understanding of cancer biology but also accelerates the development of personalized medicine by allowing for the testing of treatments on patient-derived cells in a controlled environment [6].

Fluorescence microscopy, known for its high resolution and sensitivity in visualizing sub-cellular processes, is widely employed to study biological activities in such ‘tissue-on-chip’ systems [7]. However, due to the inherent heterogeneity of these biomimetic models, it is essential to sample multiple regions within the sample to gain a comprehensive understanding of their overall characteristics and behaviors [8]. This requirement often necessitates the use of scanning systems to cover larger areas of the sample, which significantly increases both the cost and complexity associated with these microscopes, impacting both hardware and software requirements [9].

One alternative to the complex widefield scanning systems is on-chip fluorescence microscopy, where the sample is positioned in close proximity to a sensor chip that detects fluorescent signals without requiring any objective lenses [10]. This approach utilizes computational methods, such as deconvolution and compressive sensing, to effectively reconstruct images, resulting in a compact, ultra-low-cost system with a wide field of view, limited only by the sensor size, and capable of fast image acquisition [11–14]. In addition to fluorescence, on-chip microscopy based on digital holography where images are reconstructed from in-line holograms, has been extensively used in biomedical applications [15–17]. However, these on-chip microscopes often face challenges related to low sensitivity and spatial resolution [18–21].

To address these issues, we develop a cost-effective and easy-to-implement technique to enhance resolution and signal-to-noise ratio (SNR) by designing a two-dimensional microlens substrate which is placed directly beneath the sample to capture the fluorescence emission. By exciting the sample through a prism interface at an angle greater than the critical angle, we successfully rejected the excitation light after it interacted with the sample. The fluorescence emission is collected by the microlens substrate and transmitted directly to the complementary metal-oxide-semiconductor (CMOS) sensor, enabling high-resolution image formation. We theoretically optimized experimental parameters, such as the distances between the sample and microlens and microlens to sensor, to maximize the system’s sensitivity. Each microlens captures high-resolution information from its local region, allowing for the simultaneous imaging of large portions of the tumor-on-chip sample. Proof-of-concept experiments were performed by monitoring changes in oxidative stress and calcium levels at the cellular level across hundreds of cells simultaneously. This microscope was then used to monitor drug delivery and endothelial permeability on a microfluidics-based biomimetic system that replicates the tumor microenvironment.

## 2 Materials and methods

### 2.1 Materials

Human umbilical vein endothelial cells (HUVECs, CC-2519) and Endothelial Cell Growth Medium (EGM; NC0916308) were purchased from Lonza (Basel, Switzerland). The BT-20 cell line was a generous gift from Dr Amit K. Tiwari’s laboratory (University of Toledo, OH, USA). Dulbecco’s Modified Eagle Medium (DMEM), fetal bovine serum (FBS), and penicillin–streptomycin were purchased from Gibco (Thermo Fisher Scientific, USA). BT-20 cells were cultured in DMEM supplemented with 10% FBS and 1% penicillin–streptomycin. HUVECs were cultured in EGM basal medium supplemented with the EGM-2 kit according to the manufacturer’s instructions.

Propidium iodide (PI) was purchased from Invitrogen (Thermo Fisher Scientific, USA). 2′,7′-dichlorodihydrofluorescein diacetate (H_₂_DCFDA), doxorubicin (DOX), Fluo-3 (calcium indicator), calcium ionophore, calcium chloride, and fluorescein were purchased from Sigma-Aldrich (USA). Hydrogen peroxide (H_₂_O_₂_) was purchased from BDH Chemicals (VWR, USA). Dimethyl sulfoxide (DMSO, ⩾99.9% purity, molecular biology grade) was purchased from Sigma-Aldrich (USA) and used as the solvent for reagent preparation. Phosphate-buffered saline (PBS, 10× stock solution, pH 7.4) was purchased from Thermo Fisher Scientific (USA) and diluted to 1× working concentration using deionized water prior to use. Fibronectin, Matrigel, 4 kDa fluorescein isothiocyanate (FITC)-dextran, and Hanks’ balanced salt solution were purchased from Fisher Scientific (USA).

Borosilicate glass microlenses (refractive index 1.48) were purchased from Cospheric (Santa Barbara, CA, USA). Copolymer microsphere suspensions and green fluorescent polymer microparticles (1 *µ*m and 42 nm) were purchased from Thermo Fisher Scientific (USA). All solutions were prepared using ACS reagent-grade deionized water, and all chemicals and materials were used as received without further purification.

### 2.2 Preparation of microlens substrate

The imaging substrate was developed by dispersing the microspheres onto a thoroughly cleaned glass coverslip to create a uniform monolayer. The microlenses tested include borosilicate glass with diameters ranging from 150 to 180 *μ*m and a refractive index of 1.48, glass spheres with a diameter of 0.5 mm and a refractive index of 1.5, and polymeric microspheres with a refractive index of 1.59 and a diameter of 42 *μ*m (in water). Ethanol was used to moisten the dry microspheres, aiding in their uniform distribution. To prevent clumping and enhance dispersion, the coverslip was plasma treated for 50 s prior to depositing the microlenses using a benchtop high-frequency plasma generator (Electro-Technic Products Inc., Model BD-10AS, Chicago, IL, USA). It is a handheld plasma generator operated under ambient atmospheric conditions, where the plasma is generated in air without an external gas supply. Subsequently, the samples were allowed to air dry, yielding a consistent monolayer of microlenses on the coverslip.

### 2.3 On-chip microscopy

**Figure 1.**
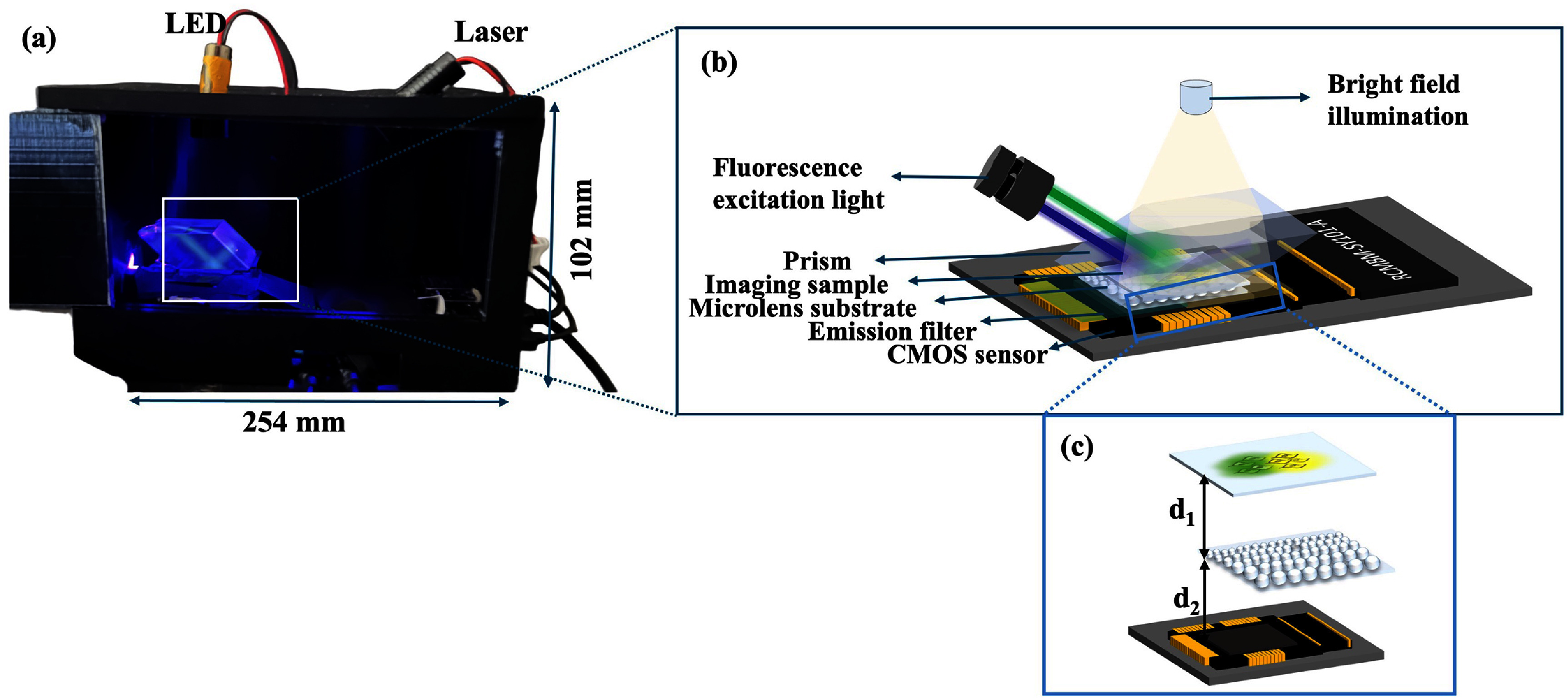
Experimental setup and geometric configuration of the microlens-assisted on-chip imaging system. (a) Photograph of the compact imaging device (10 × 4 inch) incorporating LED and laser illumination sources. (b) Schematic illustration of the imaging configuration showing both fluorescence excitation (via prism coupling) and bright-field illumination, along with the optical components including the imaging sample, microlens substrate, emission filter, and CMOS sensor. (c) Simplified geometric model defining the key system parameters: ${d_1}$, the distance between the sample and the microlens substrate, and ${d_2}$, the distance between the microlens substrate and the CMOS sensor.

The microscope was constructed utilizing custom-made 3D printed components. For the detection of fluorescence signals, a CMOS sensor array (Sony IMX219) connected via a readout cable to a Raspberry Pi single-board computer was employed. For fluorescence imaging, a blue diode laser module (Qiaoba, 445–450 nm, nominal output power 5 mW) was used as the excitation source through the side facet of the prism as shown in figure 1. In this microscopy platform, two distinct filtering mechanisms are employed concurrently to suppress excitation light and establish a dark imaging background. First, a glass prism (Edmund Optics, rhomboid shape) is used to introduce excitation light at an angle above the critical angle, inducing total internal reflection (TIR) at the bottom surface of the coverslip containing the sample. This configuration generates an evanescent field and confines excitation primarily to regions close to the sample–substrate interface. As a result, the system is inherently suited for superficial imaging of cellular structures located near the interface, rather than deep volumetric imaging of thick samples. Second, an absorption filter (Roscolux) is used to further suppress weakly scattered excitation light that does not undergo TIR. Through the combined action of these two filtering mechanisms, excitation light is effectively rejected, allowing only fluorescence emission from the sample to reach the detector plane. In addition to on-chip fluorescent imaging, bright field images of the sample can also be obtained with this platform. For bright-field imaging, an LED illumination source was positioned above the sample to provide transmitted illumination as illustrated in the figure 1.

A multimodal inverted microscope (Leica DMi8, Leica Microsystems, Germany) equipped with dark-field, phase-contrast, and fluorescence imaging modalities was used to validate microlens-assisted on-chip imaging resolution. The system was integrated with a Leica DFC3000 G digital camera (Leica Microsystems, Germany) for image acquisition.

### 2.4 Simulation of light propagation through the microlens

Finite-difference time-domain (FDTD) simulations were performed using Tidy3D (Flexcompute Inc.) [22], to characterize light propagation and focusing behavior through the microlens substrate. A 150 *µ*m-diameter borosilicate microlens (refractive index *n* = 1.48) was modeled, and a dipole emitter was positioned at sample-to-microlens distances (*d*__₁__) ranging from 77 *µ*m to 400 *µ*m, measured from the center of the microlens. For each dipole position, a planar field monitor was scanned along the optical axis on the opposite side of the microlens to determine the microlens-to-sensor distance (*d*__₂__) at which the emitted light achieved the highest photon concentration. These simulations provided spatial photon-flux distributions and axial photon-density profiles, which were used to identify the optimal focal region and to validate analytical predictions from the thick-lens model used in this study.

### 2.5 Monitoring oxidative stress in BT-20 cells treated with chemotherapeutic drugs

Proof-of-concept experiments on cells were performed using BT-20 cancer cells cultured on a glass-bottom Petri dish. The experimental setup involved seeding the cells in a modified Petri dish. To accommodate the prism for excitation, the walls of the Petri dish were cut to create a flat structure with a glass bottom in the center. The cells were then seeded in this modified Petri dish and allowed to grow. Once the BT-20 cells reached the desired confluency, they were incubated with 2’,7’-dichlorodihydrofluorescein diacetate (DCF-DA), a cell-permeant reactive oxygen species (ROS) indicator dye. DCF-DA is non-fluorescent and becomes fluorescent 2’,7’-dichlorofluorescein (DCF) after diffusion into the cell, esterase-mediated deacetylation, and oxidation by ROS. To prepare the working concentration, 1.25 mg of DCF-DA was dissolved in 0.5 ml DMSO, followed by the addition of 2 ml deionized water to make a 1 mM stock solution. Working concentrations of 10–30 *µ*M were prepared for cell staining. BT-20 cells were incubated with DCF-DA for 2 h, during which (DOX, 50 *µ*M) was added at different time points. DOX was introduced during the final phase of incubation: 15, 30, or 45 min before completion for different samples. After staining, the modified Petri dish was filled with PBS and sealed with a cover glass to maintain a stable environment. A drop of immersion oil was added to the cover glass, and a prism was positioned on top. The microlens substrate was placed underneath the glass-bottom Petri dish to collect fluorescence emission efficiently. Fluorescence from DCF was monitored at regular intervals to evaluate ROS production as a function of DOX exposure time. The same procedure was followed for hydrogen peroxide (H_₂_O_₂_) treatment to induce oxidative stress. Cell viability during imaging was assessed using 1 *µ*M PI.

### 2.6 Monitoring intracellular calcium levels in BT-20 cells

Cells were incubated with 5 *µ*M Fluo-3 AM, for one hour at 37 °C in a CO_₂_ incubator, protected from light to prevent dye excitation. Fluo-3 AM (acetoxymethyl ester) was used as a calcium indicator. The AM modification enables cell permeability; once inside the cell, it is hydrolyzed by intracellular esterases to yield the Ca^2+^-sensitive Fluo-3 dye. After incubation, cells were gently washed three times with PBS to remove any unincorporated dye, thereby minimizing background fluorescence. Calcium influx was triggered by adding 8 mM calcium chloride together with 5 *µ*M calcium ionophore (ionomycin), which helps transport calcium ions across cell membranes. After the addition of calcium chloride and the ionophore, cells were immediately imaged to observe the calcium ion influx across the membrane.

### 2.7 Establishing tumor-endothelial cells co-culture in the microfluidic device

**Figure 2.**
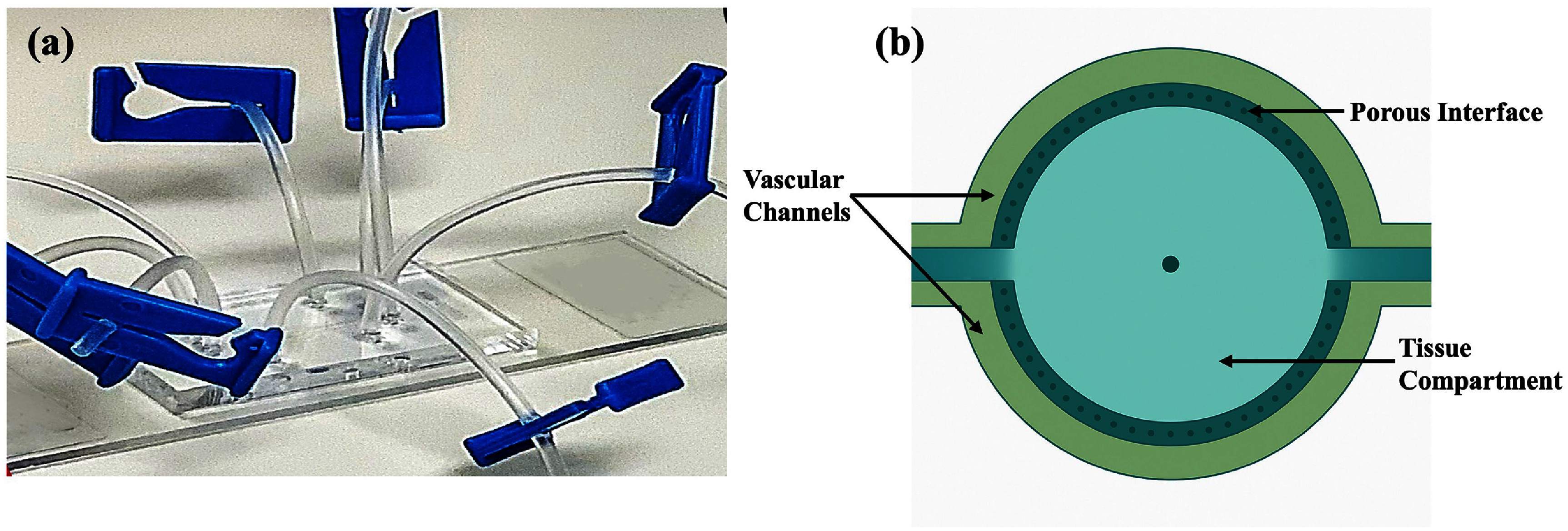
(a) The microfluidic device, fabricated from transparent PDMS with individual inlet/outlet ports connected via Tygon tubing. (b) The device features two semicircular vascular channels surrounding a central tissue compartment, each independently perfused. The vascular channels and tissue compartment are separated by a porous interface consisting of a microfabricated porous barrier that enables co-culture interactions between tumor cells and endothelial cells, facilitating information and mass exchange.

A polydimethylsiloxane-based microfluidic device (SynVivo IMN-2 Radial Idealized Co-culture Network Chips) with three channels—two semicircular vascular channels (200 *µ*m width) surrounding a central tissue compartment (1.8 mm diameter)—was utilized to establish the primary TME model. The channels were separated by microfabricated porous interfaces with a 50 *µ*m travel distance. The microfluidic device contained 8 inlet/outlet ports connected to the three channels and was bonded to a coverslip, facilitating real-time imaging and visualization of cellular interactions within the channels. Briefly, the device consisted of three channels: two semicircular vascular channels where endothelial cells were seeded and one circular tissue compartment for seeding cancer cells, all interconnected by porous interfaces to enable media and nutrient exchange. The configuration of the microfluidic chip is shown in figure 2. For co-culture creation, HUVECs between passages 1 and 5, and BT-20 cells up to passage 30, were utilized. HUVECs were grown in T-75 flasks with EGM basal media supplemented with the EGM2 kit, and BT-20 cells were cultured in T-75 flasks in DMEM media supplemented with 10% FBS and 1% Penicillin-Streptomycin. Both cell types were maintained until ready for seeding in the microfluidic devices, and contamination checks were performed before their use in assays.

The microfluidic device was prepared using a previously established protocol [23, 24]. Briefly, the microfluidic device was rinsed with deionized water and degassed to eliminate any trapped air bubbles within the channels. The vascular channels were then coated with fibronectin diluted in PBS to a final concentration of 200 *µ*g ml^−1^. Following fibronectin coating, the device was incubated at 37 °C for 1 h to allow a thick layer to form within the vascular channels. HUVECs were then seeded in the vascular channels at a final concentration of 25 million cells ml^−1^ using an automated syringe pump and cultured under a static flow scheme for 4 h before starting the dynamic flow scheme within the vascular channels. After 24 h of HUVEC seeding, BT-20 cells (BT-20) were seeded in the tissue compartment at a concentration of 15 million cells ml^−1^, resuspended in Matrigel. The device seeded with BT-20 cells was incubated at 37 °C for another 24 h, allowing the primary TME model to form, including both endothelial and BT-20 cells.

Media perfusion in the vascular channels was maintained using the Harvard Apparatus PHD Ultra multirack system. The flow scheme consisted of a constant flow rate of 1 *µ*l min^−1^ for 15 min, followed by a 4 hr delay. This two-step process was repeated six times, after which a continuous flow rate of 0.1 *µ*l min^−1^ was maintained until the end of the experiment. After 72 h of HUVEC seeding and 48 h of BT-20 cell seeding, the device was used for various assays, including permeability assays, calcium imaging, and nanoparticle diffusion from one channel to another.

## 3 Result and discussion

### 3.1 Optimization of microlens parameters for enhanced performance

**Figure 3.**
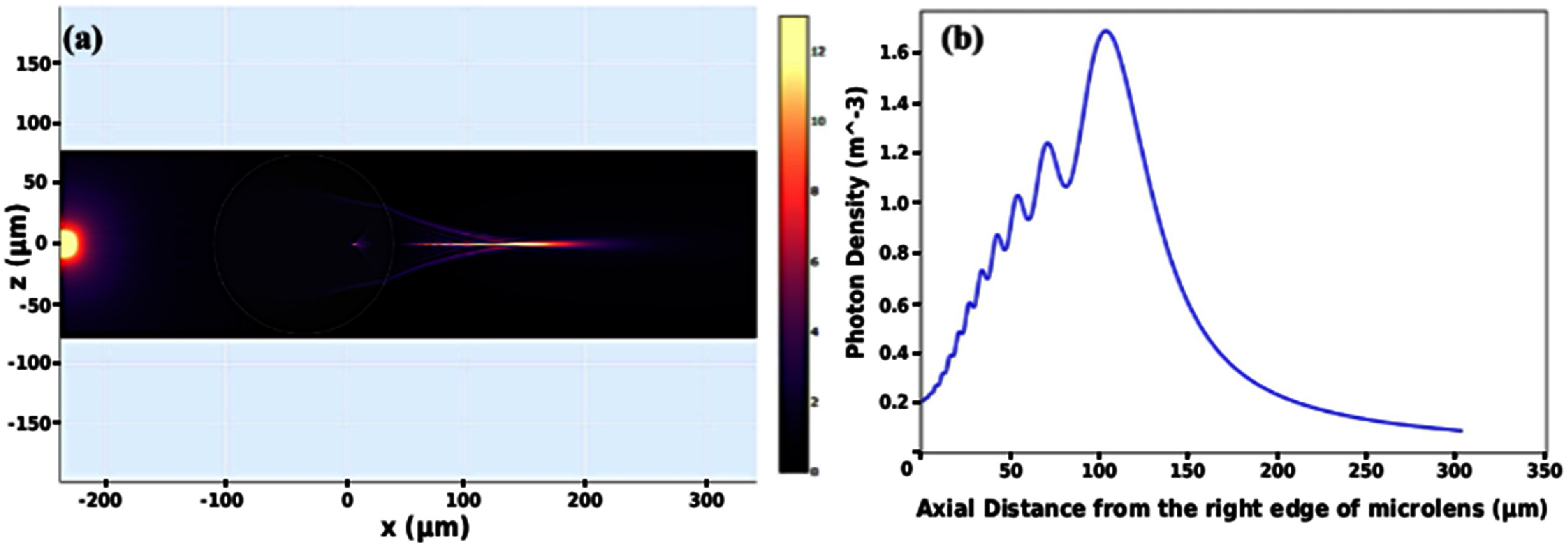
(a) FDTD simulation showing the light collection and focusing behavior of a 150 *µ*m microlens for an isotropic dipole emitter positioned 200 *µ*m from the center of the microlens. The color map represents the normalized photon flux intensity distribution, illustrating photon concentration near the focal region and delivery toward the sensor plane. (b) Simulated photon-density profile as a function of the axial distance measured from the right edge of the microlens. A maximum photon density is obtained when the sensor plane coincides with the real image plane of the microlens, confirming optimal coupling conditions for maximizing SNR in the on-chip fluorescence microscope.

The photon density on the CMOS sensor is essential for maximizing the SNR and thereby enhancing the sensitivity of the imaging system. The photon density in each pixel is influenced by the efficiency of the lens in collecting fluorescent photons and the area over which the light is focused. These factors are governed by the sample to microlens and microlens to sensor distance. For objects located beyond the focal point of the microlens, the photon density profile along the axis, as demonstrated by the FDTD simulations, using Tidy3D (Flexcompute Inc.) [22], is shown in figure 3. It should be noted that the simulations model the propagation and collection of fluorescence emission after excitation, with the aim of optimizing ${d_1}$ and ${d_2}$ for maximum photon collection efficiency, and do not explicitly include the TIR excitation geometry or the associated evanescent excitation field. To establish guidelines for the relationship between the distances from the sample to the microlens and from the microlens to the sensor, we developed a semi-analytical solution based on thick lens to calculate the amount of light collected and the area over which the light is distributed. The fraction of fluorescence signal collected by the microlens was calculated by determining the solid angle subtended by the microlens with the dipole emitter. In addition, ray-tracing based on thick-lens vergence theory was used to compute how the collected rays propagate through the microlens and reach the sensor plane. A function modeling the photon density based on both variables was determined and is presented in equation (1), where *D* is the photon density, *d*_1_ and *d*_2_ are the distances of the sample and sensor from the microlens, r is the microlens radius, and f is the focal length of microlens

\begin{equation*}D=\frac{1-\frac{d_1}{\sqrt{d_1^2+r^2}}}{2\pi\left[\left(d_2-\left(\frac{1}{f}-\frac{1}{d_1}\right)^{-1}\right)\left(\frac{r}{f}-\frac{r}{d_1}\right)\right]^2}\end{equation*}

**Figure 4.**
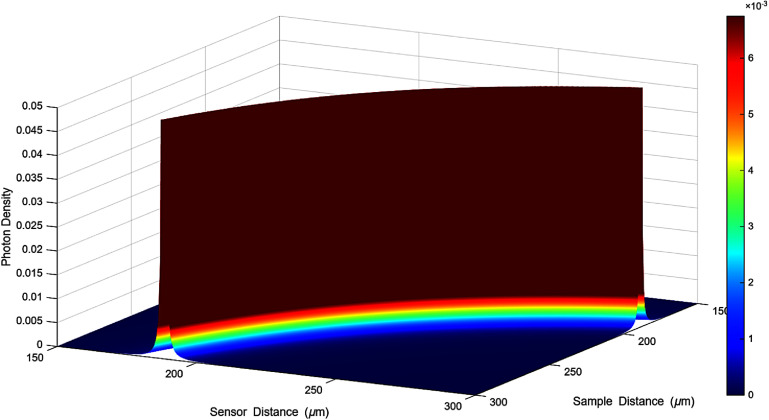
Simulated photon density as a function of the sample-to-microlens and microlens-to-sensor distances. The plot shows a narrow ridge where the photon density reaches its maximum value, indicating the configuration in which the sensor aligns with the real image plane of the microlens. This maximum represents the point at which the microlens delivers the highest photon concentration to the sensor, defining the optimal geometry for maximizing signal collection.

The photon density is shown as a function of the sample-to-microlens and microlens-to-sensor distances in figure 4. The photon-density distribution in figure 4 is based on a geometrical optics model using a thick-lens approximation and does not include diffraction, aberrations, or pixel integration effects. The narrow ridge corresponds to geometries that maximize photon collection efficiency and reflects the sensitivity of the system to alignment. In practice, a finite range of configurations around this region provides comparable photon collection. The photon density reaches its maximum when the real image plane is aligned with the sensor plane. The relationship between the sensor-to-microlens distance (*d*__₂__) and the sample-to-microlens distance (*d*__₁__) required to achieve maximum photon density is shown in figure S1. Here, the blue curve represents the analytical model predictions, while the orange points denote the numerical FDTD simulation results.

The numerical results follow the general trend predicted by the analytical thick-lens model, particularly at larger sample-to-microlens distances. However, as the sample moves closer to the microlens, the deviation between the analytical and FDTD results becomes more pronounced. This difference is expected because the analytical model is based on a geometrical thick-lens approximation and does not account for diffraction or aberration effects present in the ball-lens geometry. In particular, spherical aberration causes marginal and paraxial rays to focus on different axial positions, broadening the effective point spread function (PSF) and limiting intrinsic spatial resolution. Therefore, although the microlens substrate improves photon collection efficiency and increases the detected signal and SNR, its primary benefit is enhanced feature visibility and contrast rather than a direct elimination of aberration-limited resolution. The influence of spherical aberration may be reduced by applying computational image restoration.

### 3.2 Imaging fluorescent microparticles

**Figure 5.**
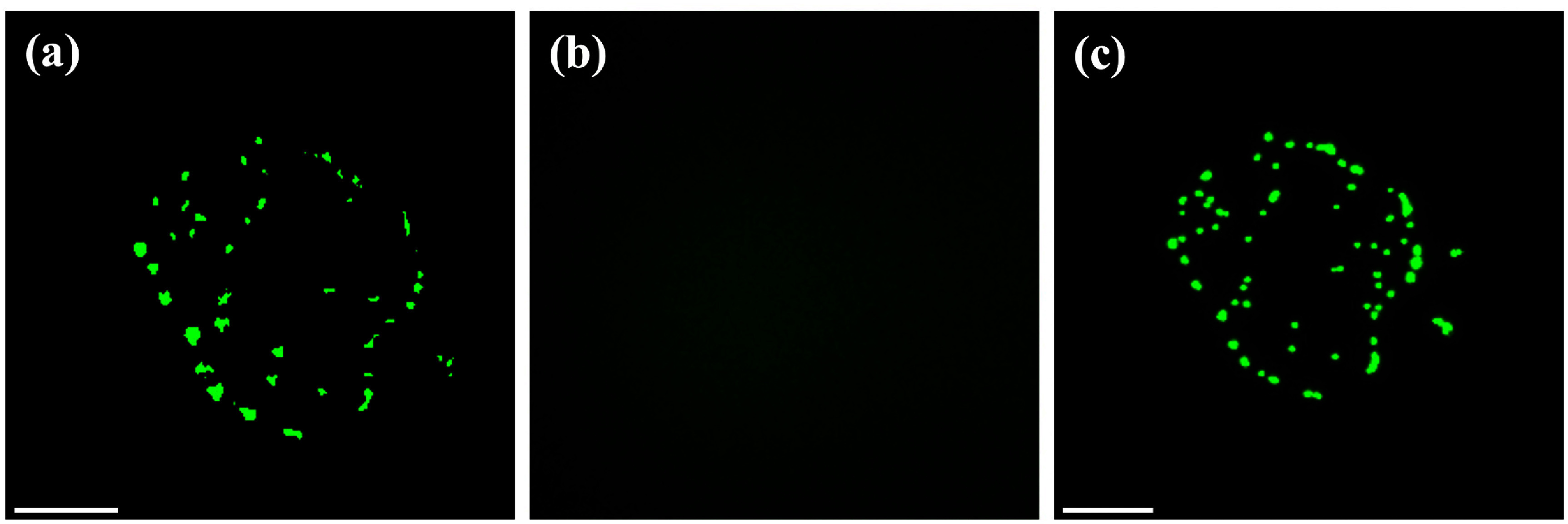
Imaging of 1 *μ*m fluorescence particle (a) with (b) without microlens (c) conventional fluorescence microscopy with 10x objective, NA = 0.25. The scale bar is 40 *μ*m.

We first evaluated the functionality of microlens substrate-integrated fluorescence microscopy by imaging 1 *µ*m fluorescent particles as shown in figure 5. Incorporating the microlens array resulted in substantially brighter particle signals, making them easier to detect compared to imaging without the microlenses, where fluorescence appeared faint and close to background levels. This improvement arises from the microlenses concentrating emitted photons onto the sensor, thereby increasing the effective photon flux collected from each emitter. The images were further improved by standard image processing techniques. The raw images were processed by first suppressing background noise using a combination of white top-hat filtering with Gaussian background subtraction, difference-of-Gaussians filtering, and mean filtering. The images were then binarized using Otsu’s adaptive thresholding and refined through morphological operations, followed by watershed segmentation to separate adjacent features. Image sharpness was enhanced by deconvolution and subsequent unsharp masking. Row images are represented in figure S3. Representative images acquired using a conventional fluorescence microscope with a 10X objective are also shown in figure 5 for visual comparison of fluorescence appearance.

To evaluate feature localization and image sharpness, fluorescence intensity profiles were extracted from representative 1 *µ*m fluorescent particles imaged using the microlens-assisted on-chip system and the conventional fluorescence microscope. Because the particle diameter is comparable to or larger than the expected spatial resolution of the on-chip platform, these particles cannot be considered point sources for accurate PSF characterization. Therefore, the Gaussian-fitted profiles should not be interpreted as the true optical PSF. Instead, they provide an apparent particle-image width that reflects the combined contribution of the finite particle size and the imaging system response. Using this analysis, the microlens-assisted on-chip system produced localized particle profiles with an apparent width of approximately 1.27 *µ*m, while the conventional fluorescence microscope produced a narrower apparent profile of approximately 1 *µ*m. These results support the conclusion that the microlens substrate improves particle visibility and signal localization compared with imaging without the microlens substrate. A detailed line-profile analysis is provided in supplementary figure S3. Furthermore, bright-field on-chip imaging of breast cancer cells (MDA-MB-231) was performed under identical conditions, as shown in figure S4. Regions aligned with microlenses exhibit enhanced contrast and improved delineation of cellular structures, where individual cell boundaries and internal features become more clearly visible. In contrast, regions without microlens coverage appear diffuse, and cellular structures are not clearly distinguishable. This spatially varying image quality within the same field of view further illustrates the role of the microlens in improving effective resolution and feature visibility. Although the spatial resolution of the on-chip platform is improved, it remains constrained by system parameters including the effective numerical aperture, the pixel size of the CMOS sensor, and optical aberrations introduced by the microlens geometry.

Although the spatial resolution of the on-chip platform is improved, it remains constrained by system parameters including the effective numerical aperture, the pixel size of the CMOS sensor, and optical aberrations introduced by the microlens geometry.

To evaluate further the influence of the microlens substrate on detection sensitivity, serial dilutions of FITC dye were prepared, ranging from 10 pM to 700 nM. Samples were imaged under consistent illumination settings, both with and without the microlens array. Fluorescence intensity was quantified for each concentration under identical imaging conditions. We observe the fluorescence intensity increased steadily with rising FITC concentration beyond the limit of detection for both configurations, until reaching saturation at higher concentrations. A key difference, however, is that the microlens-assisted system consistently generated higher fluorescence signals at lower concentrations. Notably, at low concentrations (⩽10 pM), fluorescence signals acquired without the microlens substrate were indistinguishable from background noise, whereas clear and quantifiable signals were observed when the microlens array was used. This improvement results from the microlens’s capability to collect and focus isotropically emitted photons onto the sensor, thereby increasing the effective photon density and augmenting the SNR. A graph illustrating fluorescence intensity versus FITC concentration, with and without the microlens substrate, is presented in figure S2.

To further validate imaging performance beyond fluorescence intensity measurements alone, we quantified the signal-to-background ratio SNR using a 500 nM FITC solution imaged under identical conditions with and without the microlens substrate. The signal-to-background ratio SNR was calculated by comparing the mean fluorescence signal to the background noise level in each configuration. The microlens-assisted configuration exhibited approximately 2–2.5-fold higher signal-to-background ratio SNR than the configuration without the microlens substrate, indicating that the microlens significantly improves fluorescence detectability by enhancing photon collection efficiency and increasing signal-to-background noise. This result supports the conclusion that the microlens substrate improves not only signal intensity but also the reliability of fluorescence detection in the on-chip imaging platform.

### 3.3 Imaging of BT-20 cells for anti-cancer drug screening

**Figure 6.**
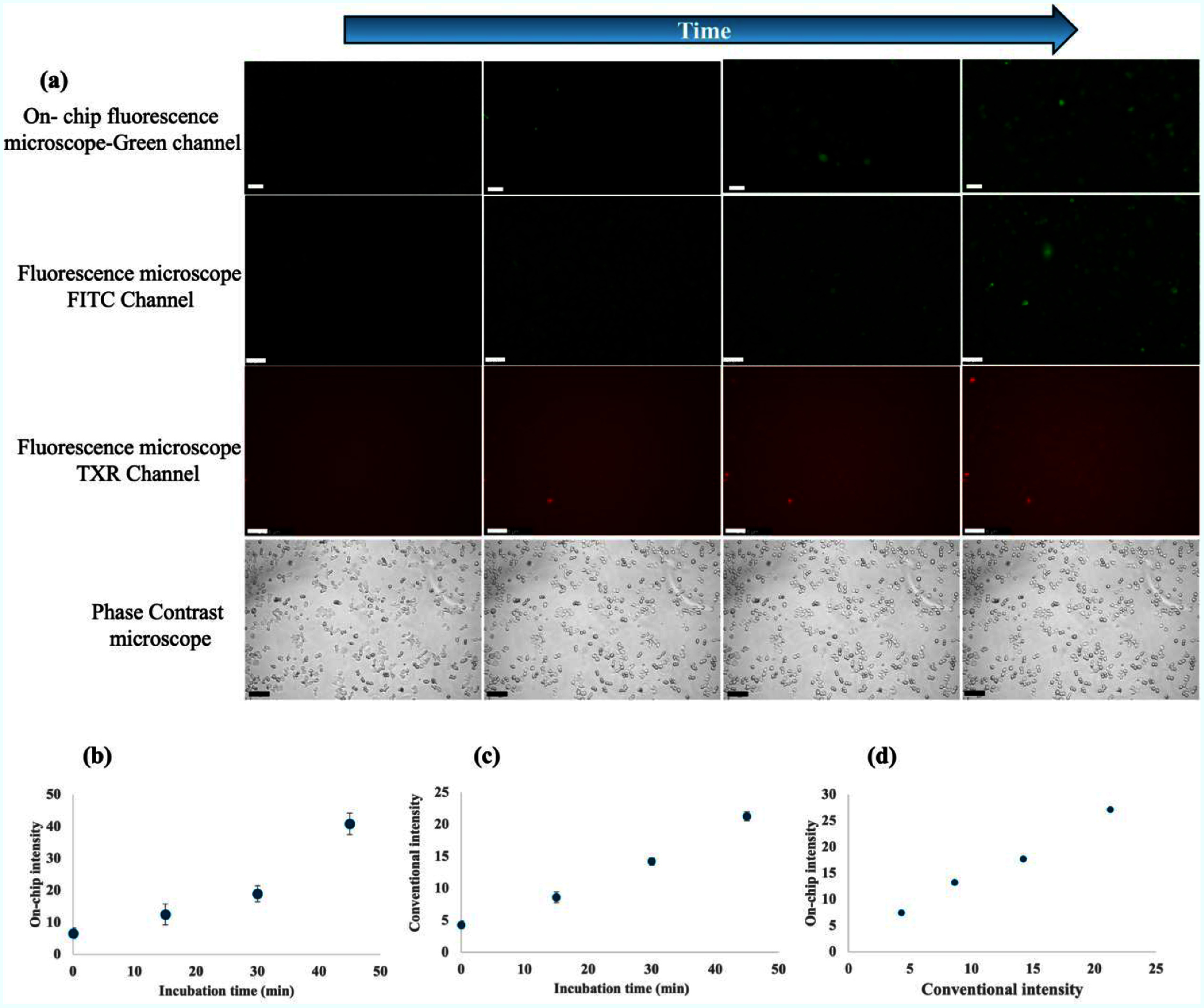
(a) Time series images of cells treated with 50 *μ*M DOX in the first row; on-chip fluorescence microscope. Second row: fluorescence microscope in FITC channel to measure DCF excitation. Third row: fluorescence microscope in TXR channel to monitor cell viability by staining cells with 1 *μ*M PI. Fourth row: phase contrast microscope, with 10x objective. (b) Intensity graph using (b) on-chip fluorescence microscopy, (c) using a 10x objective with conventional fluorescence microscopy. (d) Correlation plot comparing fluorescence intensities measured using the conventional microscope and the on-chip microscope, showing strong linear agreement (Pearson’s *r* = 0.99). Error bars represent standard deviation (SD) calculated from 20–30 cells across 3 independent experiments. The scale bar is 100 *μ*m.

**Figure 7.**
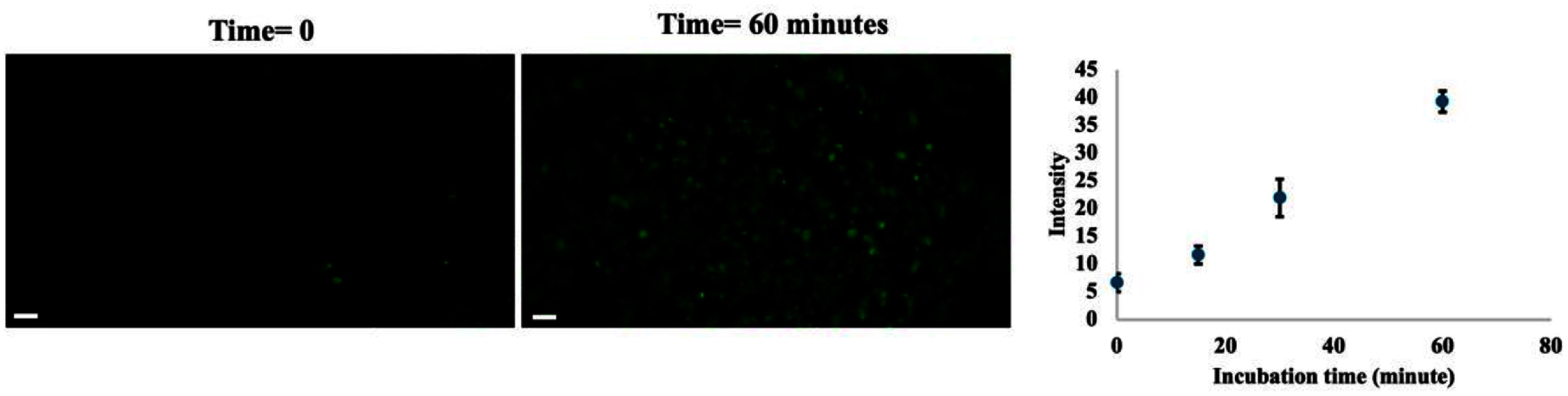
Time series images of cells treated with 1 mM H_2_O_2_ using on-chip fluorescence microscope and intensity graph versus time. the corresponding plot shows fluorescence intensity as a function of incubation time, demonstrating the time-dependent rise in ROS levels. Error bars represent standard deviation (SD) calculated from 20–30 cells across 3 independent experiments. The scale bar is 100 *μ*m.

Building on the improved detection sensitivity, we evaluated the performance of the on-chip fluorescence microscope in a biologically relevant assay by imaging BT-20 breast cancer cells treated with a chemotherapeutic agent. DOX, a DNA-intercalating chemotherapeutic that intercalates into genomic DNA and undergoes redox cycling to generate superoxide and hydrogen peroxide, thereby elevating intracellular oxidative stress, eventually leading to apoptosis [25, 26]. Monitoring DOX-induced oxidative stress is critical for assessing therapeutic efficacy and cell viability. BT-20 cells were incubated with both DCF-DA and DOX. The incubation time for DOX was varied, with cells being incubated with 50 *µ*M DOX for 0, 15, 30, 45, and 60 min to monitor time-dependent oxidative stress responses. We observed that the fluorescence intensity increased with longer DOX incubation times. This increase in intensity is attributed to the fact that DOX induces oxidative stress in the cells, leading to the production of ROS. As the cells were incubated with DOX for longer durations, more ROS were generated, which in turn increased the fluorescence signal of the DCF-DA probe (figure 6(a) first row). In addition, the intensity changes were monitored using conventional fluorescence microscopy, which showed a similar trend to the results obtained with the on-chip fluorescence microscope (figure 6(a), second row). Figures 6(b) and 6(c) present the fluorescence intensity of BT-20 cells over time measured using both the on-chip fluorescence microscope and a conventional fluorescence microscope, showing consistent temporal trends in the increase of DCF signal with more prolonged DOX incubation. Conventional fluorescence imaging was done in FITC channel (Ex. 495 nm and Em_max_: 520 nm). To further evaluate the agreement between the two imaging modalities, an additional plot was generated with the intensity measured using the conventional fluorescence microscope on the *x*- axis and the corresponding intensity measured using the on-chip system on the *y*-axis. The data showed a strong linear relationship between the two measurements, with a Pearson’s correlation coefficient of 0.99, indicating excellent consistency between the intensity values obtained from the conventional microscope and the on-chip device. To assess cell viability during the experiment, the cells were also stained with 1 *µ*M PI. The results, shown in figure 6(a) (third row), indicate that the cells remained viable throughout the imaging period. Imaging with PI was performed using a conventional fluorescence microscope in the Texas Red channel (Ex: 595 nm, and Em_max_: 615 nm), confirming cell viability during the entire experiment. Subsequently, we applied the same intracellular ROS detection protocol using hydrogen peroxide as the oxidative stress inducer. Although hydrogen peroxide (H_₂_O_₂_) is generated during normal cellular metabolism and plays an essential role in redox signaling, excessive accumulation overwhelms cellular antioxidant defenses, causes oxidative damage, and activates stress-induced cell-death pathways [27–29]. BT-20 cells were first stained with DCF-DA and then exposed to 1 mM H_₂_O_₂_ for 15, 30, and 60 min. Hydrogen peroxide directly diffuses into cells and generates hydroxyl radicals via Fenton-type reactions, rapidly elevating intracellular oxidative stress [30, 31]. As shown in figure 7, Fluorescence intensity increased over time in a manner that reflects the same trends observed with DOX treatment.

**Figure 8.**
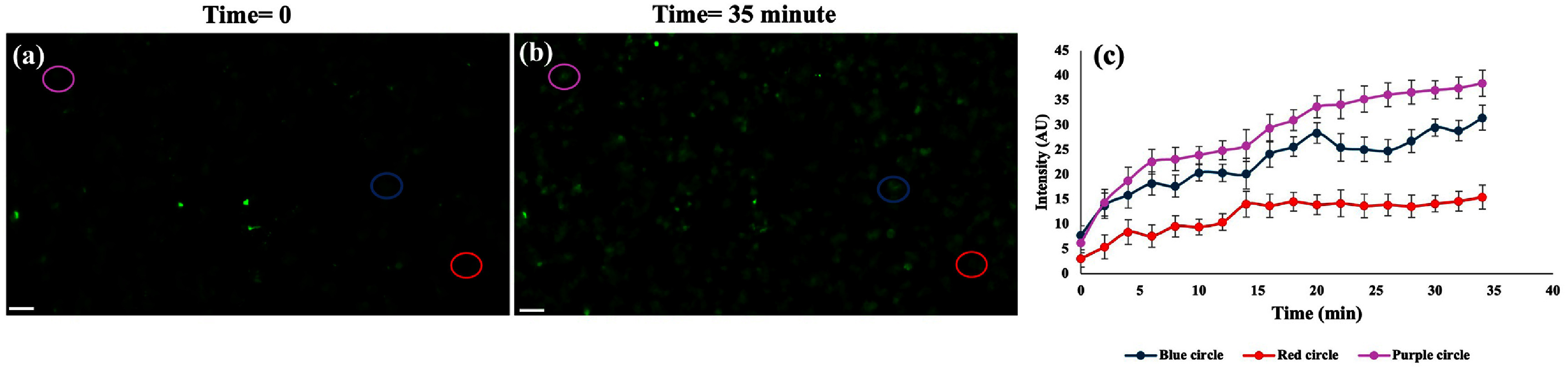
(a) and (b) Representative fluorescence images of BT-20 cells at Time = 0 and 35 min after treatment with calcium chloride (8 mM) and calcium ionophore (5 *µ*M). The analyzed cells are marked by purple, blue, and red circles. (c) Fluorescence intensity profiles of the selected cells plotted versus time, demonstrating a gradual increase in intracellular calcium after ionophore treatment. The fluorescence signal is reported as intensity (AU). Error bars indicate the standard deviation (SD) of pixel intensities within each selected cell region at each time point. Scale bar: 100 *µ*m.

Having validated our platform for monitoring gradual, drug-induced oxidative stress, we next sought to demonstrate its capability for capturing fast, transient biochemical signals. In particular, we turned to calcium imaging, a critical readout of real-time cellular signaling, because intracellular Ca^2+^ signaling is essential for neuronal function, synaptic communication, and cell survival, and dysregulation of Ca^2+^ homeostasis is a well-established hallmark of neurodegenerative diseases, including Alzheimer’s disease, Parkinson’s disease, and amyotrophic lateral sclerosis, where disrupted Ca^2+^ handling contributes to neuronal dysfunction and degeneration [32–35]. To this end, BT-20 cells were incubated with the cell-permeable indicator Fluo-3 AM, which, following intracellular de-esterification, emits fluorescence proportional to free Ca^2+^. We then perfused the cells with 8 mM CaCl_₂_ in the presence of 5 *µ*M calcium ionophore to facilitate instantaneous Ca^2+^ influx across the plasma membrane. Real-time imaging captured a rapid and pronounced surge in fluorescence intensity, reflecting the kinetics of intracellular calcium elevation. Figure 8 depicts the time course of these fluorescence changes in a representative BT-20 cell, confirming that our microlens-enhanced on-chip microscope can sensitively track subcellular signaling dynamics. To further investigate variability across individual cells within the wide field of view, in figure 8(b) we performed a single-cell intensity analysis by tracking fluorescence changes over time for representative cells (indicated by white, yellow, and blue circles) located in different regions of the imaging area. They exhibit a low baseline signal at time 0, whereas at 35 min, an apparent increase in brightness is visible across the cytoplasm.

After demonstrating that the on-chip microscope could reliably capture dynamic signaling events at the cellular level, we next applied the system to biomimetic models. For proof-of concept experiments we choose a microfluidic-based biomimetic platform that simulates the endothelial barrier of tumors, as they have been widely used for replicating tissue microenvironments and capturing dynamic cellular responses. We utilize the on-chip microscope to study the endothelial barrier permeability under controlled flow conditions. Assessing tracer and nanoparticle transport across an endothelium-lined channel provides critical insights into drug delivery mechanisms and barrier function in microphysiological systems.

**Figure 9.**
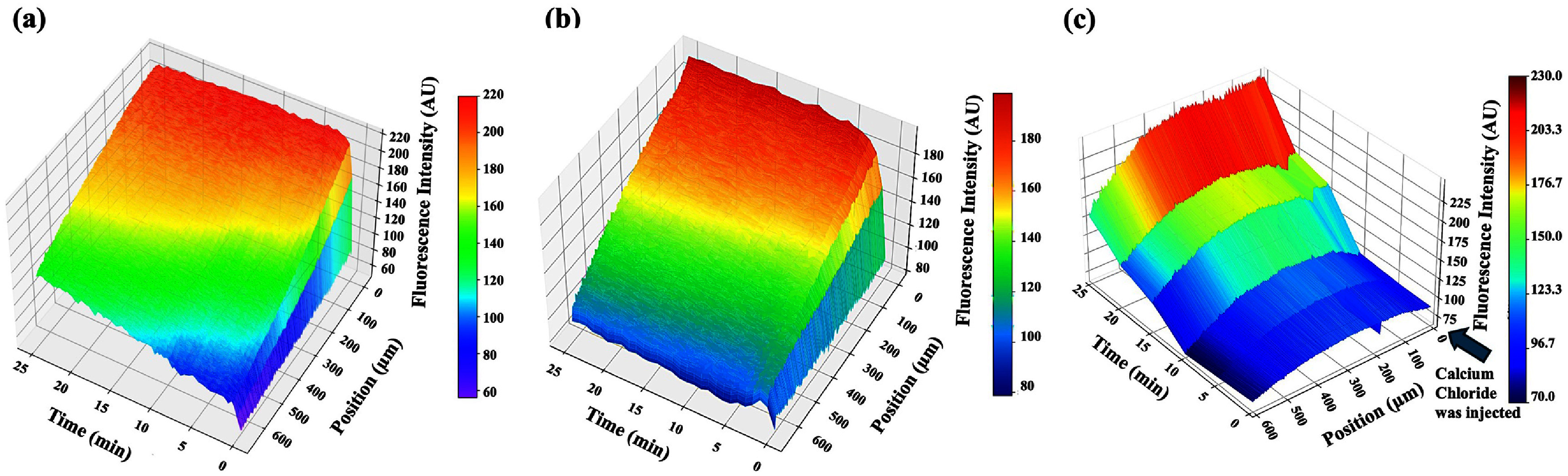
Fluorescence-based analysis of diffusion and intracellular signaling in the microfluidic tumor–endothelial co-culture platform. Time-dependent diffusion of (a) FITC–4 kDa dextran from the vascular channel into the tissue compartment across the endothelial barrier under flow. (b) 42 nm fluorescent nanoparticles under identical flow conditions, showing reduced permeability compared to small-molecule dextran. (c) CaCl_₂_ injected through the vascular channel into the tissue compartment across the endothelial barrier under continuous flow.

In this assay, we first infused FITC-labeled 4-kilodalton dextran (2.5 mM) through the vascular channel at 1 *µ*l min^−1^ using an automated syringe pump, continuously recording fluorescence intensity over 30 min to track dye extravasation into the tissue compartment (figures 9(a) and S5). A steady increase in fluorescence intensity was observed within the tissue region, indicating gradual diffusion of the dye through the endothelial barrier. The ongoing rise in intensity confirmed that the endothelial monolayer allowed the passage of small solutes, consistent with expected paracellular transport behavior in microvascular systems. Next, we assessed the barrier permeability relevant to nanoparticles, which has been extensively used as anti-cancer drug carriers [36–38]. We introduced 42 nm fluorescent nanoparticles (Ex. 495 nm and Em_max_: 520 nm) under identical flow parameters and quantified their transport profile (figures 9(b) and S6). Compared to the 4 kDa dextran, nanoparticle penetration across the endothelial channel was substantially slower and more restricted, as indicated by lower fluorescence intensity in the tissue compartment over time. This result confirms size-dependent transport behavior, demonstrating that the endothelial barrier effectively limits nanoparticle extravasation relative to small molecules. After quantifying the transport of small molecules and nanoparticles across the endothelial layer, we directed our focus to the sensing of changes in intracellular chemical analytes within the tumor core in a biomimetic environment. For this purpose, we imaged calcium ions as they serve as essential secondary messengers in endothelial signaling to regulate vascular tone, barrier integrity, and inflammatory responses. BT-20 cells in the central chamber were loaded with Fluo‐3, and calcium ionophore plus calcium chloride were perfused via the vascular inlet. Time-lapse imaging captured the onset and progression of calcium influx across the endothelial layer is shown in figure 9(c). In this biomimetic co-culture system, we observed a clear increase in intracellular fluorescence intensity in the cells following introduction of calcium ionophore and CaCl_₂_, indicating rapid Ca^2+^ entry into the tumor compartment (figures 9(c) and S7). The increase in fluorescence originated near the vascular interface and propagated inward, demonstrating that the platform can capture diffusion-driven ionic signaling across the endothelial barrier.

Overall, these experiments offer quantitative permeability profiles for molecules, nanoparticles, and ions across an endothelial monolayer under flow. By analyzing time-lapse intensity profiles, we qualitatively compared barrier integrity and transport behavior for molecules, nanoparticles, and ions under flow conditions. Such data enable the rational selection of carrier size and dosing regimens in tissue-on-chip models, offering a metric for comparing barrier function under different physiological or pathological conditions.

The demonstrated applications collectively validate the performance and versatility of the proposed on-chip imaging platform. Despite its simplified design, the system enables reliable multimodal imaging and quantitative analysis, confirming its effectiveness for a range of biological and microfluidic investigations. Importantly, the overall system cost is estimated to be approximately $127–$147, as detailed in table S1 (supplementary section). This low-cost implementation, combined with the use of commercially available components and straightforward fabrication methods, highlights the potential of the platform for deployment in resource-limited settings and for applications requiring accessible and scalable imaging solutions.

## 4 Conclusion

In this study, we presented and validated a new, low-cost on-chip fluorescence microscopy system enhanced by a two-dimensional microlens array integrated directly onto a CMOS sensor. This innovative setup significantly improved sensitivity, spatial resolution, and imaging throughput, providing an accessible and practical alternative to traditional fluorescence microscopes that often depend on costly optics and mechanical scanning stages to cover a large field of view. The use of a prism-based lateral excitation method helped effectively reject excitation light through TIR, significantly boosting the SNR. Analytical calculations combined with FDTD simulations optimized parameters to maximize photon density, identifying the best sample-to-microlens and microlens-to-sensor distances. This demonstrated precise control over photon density for achieving optimal sensitivity. The platform’s effectiveness was demonstrated through biologically relevant assays. Monitoring oxidative stress induced by DOX and hydrogen peroxide in BT-20 breast cancer cells highlights the importance of accurately detecting intracellular ROS, which is essential for assessing drug efficacy and cellular toxicity in therapeutic contexts. Rapid intracellular calcium dynamics were successfully visualized using the calcium-sensitive dye Fluo-3, highlighting the microscope’s capability to track transient biochemical signals vital for understanding cellular signaling pathways and investigating neurodegenerative diseases. Furthermore, the microscope’s versatility was showcased through a microfluidic-based biomimetic system designed to replicate TME. Evaluating endothelial barrier permeability provided crucial quantitative profiles for small molecules, nanoparticles, and ions, enabling improved insights into drug delivery mechanisms and endothelial health under controlled physiological conditions. This capability positions the platform effectively for high-throughput drug screening, biomimetic tissue modeling, and detailed investigations of cellular interactions.

Therefore, this on-chip fluorescence microscopy platform meets essential needs for portability, affordability, high resolution, and wide-field imaging in biomedical research. Its successful application in multiple bioassays highlights its potential as an accessible tool in biomedical diagnostics, drug discovery, and basic cellular biology research.

## Data Availability

All data that support the findings of this study are included within the article (and any supplementary files).
